# Relationship of corneal hysteresis and optic nerve parameters in healthy myopic subjects

**DOI:** 10.1038/s41598-017-15386-6

**Published:** 2017-12-13

**Authors:** Kunliang Qiu, Xuehui Lu, Riping Zhang, Geng Wang, Mingzhi Zhang

**Affiliations:** 1 Joint Shantou International Eye Center of Shantou University and The Chinese University of Hong Kong, Shantou, Guangdong Province The People’s Republic of China; 2 Department of Ophthalmology, University of Groningen, University Medical Center Groningen, Groningen, The Netherlands

## Abstract

The association between corneal biomechanical properties and glaucoma is an area of much interest. We determined the relationship between corneal hysteresis (CH) and optic nerve parameters in healthy myopic subjects in the current study. CH was measured with Reichert Ocular Response Analyzer in 108 eyes from 108 healthy myopic subjects. All subjects received retinal nerve fiber layer and optic disc imaging Cirrus HD-OCT, GDx ECC, and Heidelberg Retina Tomograph II. None of the tested optic nerve parameters showed statistical significance with CH by using correlation analysis. For RNFL parameters, there was a negative but not statistically significant correlation between CH and average RNFL thickness obtained with OCT (r = −0.15, p = 0.13). For optic disc parameters, there was a negative but not statistically significant correlation between CH and rim area measured with OCT (r = −0.10, p = 0.29). The current study did not find any statistically significant relationship between CH and optic nerve parameters as measured by all three imaging modalities in healthy myopic eyes. Therefore, the relationship observed previously in glaucoma subjects is likely coming to fruition as optic nerve damage is caused by the disease.

## Introduction

The association between corneal biomechanical properties and glaucoma is an area of much interest^1–3^. The Ocular Response Analyzer (ORA) has been developed to analyze *in vivo* corneal biomechanical properties including the corneal hysteresis (CH) and the corneal resistance factor (CRF)^4^. It is speculated that corneal biomechanical properties could reflect structural vulnerabilities of the entire eye that increase its susceptibility to glaucoma. Previous studies have reported that lower CH is associated with both structural and functional damage in glaucoma^2,5^. Moreover, it has been shown, in recent studies, that CH measurements are significantly associated with risk of glaucoma progression^6,7^. However, while the association has been examined in glaucomatous eyes, there is limited and conflicting data regarding the relationship between CH and optic nerve parameters in healthy subjects. In a population-based study, neither CH nor CCT was found to correlate with measures of optic disc cupping^8^. In contrast, CH was reported to positively correlate with rim area and RNFL measurement in 5134 British subjects^9^. Thus, controversies exist regarding the association between corneal biomechanical properties and structural measurements of the optic nerve.

Myopia is a common ocular disorder which has been shown to be one of the risk factor for primary open angle glaucoma^10,11^. Although the underlying mechanism between myopia and risk of glaucoma is not fully understood, it has been suggested that eyes with a long axial length have a greater deformability of the lamina cribrosa which might contribute to a higher susceptibility to glaucomatous damage^12^. Furthermore, lamina cribrosa defects have been reported to be associated with glaucomatous damage in myopic eyes^13,14^ As the sclera and cornea are composed of an integrated connective tissue layer, alteration of CH (a quantitative measurement of cornea deformation) may reflect the change of lamina cribrosa. Previously, CH has been found to be lower in myopic eyes, glaucomatous eyes, and in patients with unilateral nonarteritic anterior ischemic optic neuropathy^2,5,15–17^.All these findings suggest that decrease of CH could probably reflect structural weakness in the lamina cribrosa which increase its susceptibility to glaucoma in myopic eyes. Therefore, evaluation of the fundamental relationship between corneal biomechanical properties and optic nerve morphology in healthy myopic eyes may provide an insight into the increased susceptibility to glaucoma in myopia subjects.

In view of the clinical importance of corneal hysteresis and the controversies about the relationships between corneal hysteresis and optic nerve parameters, we aimed to investigate the association of corneal hysteresis with the structural measurements of the optic nerve by using 3 commonly used imaging devices in healthy myopic subjects.

## Results

**Figure 1:**
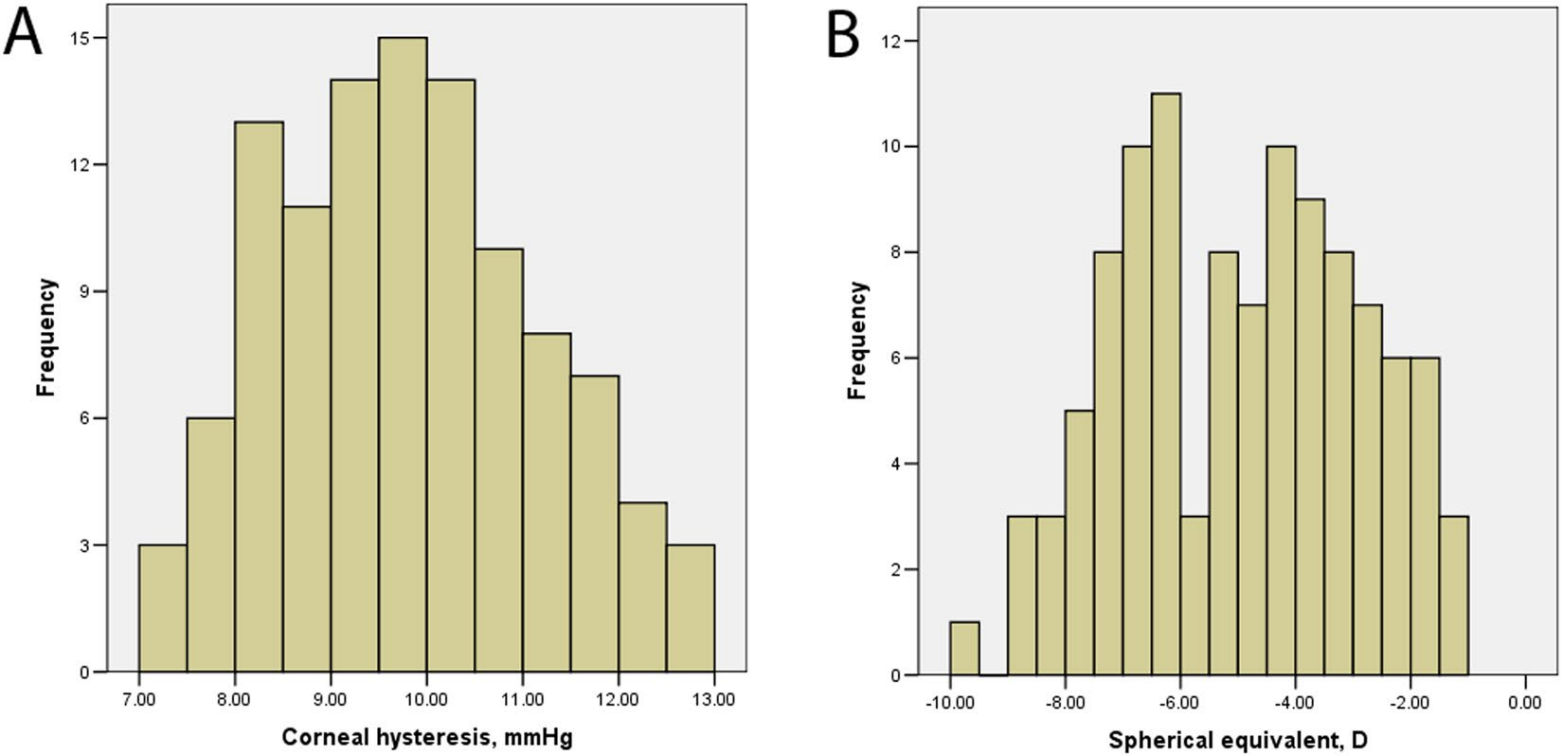
Histogram of corneal hysteresis (**A**) and spherical equivalent (**B**) of all included eyes.

Eight subjects were excluded because of unreliable visual field tests (5 subjects) and poor OCT scan quality (3 subjects). As a result, we included 108 eyes from 108 subjects (66 females and 58 right eyes). Table 1 shows the demographics of the study population. The mean refractive error and axial length were −4.91 ± 2.03 D (range, −9.63 to −1.00 D) and 25.60 ± 1.04 mm (range, 22.62 to 28.77 mm), respectively. The mean CH and CCT was 9.77 ± 1.35 mmHg (range, 7.03 to 12.73 mm) and 540.9 ± 28.8 μm (range, 444 to 612 μm), respectively. Figure 1 displays the distribution of CH and refractive status across all subjects. CH was significantly associated with axial length and refractive error(r = −0.20, p = 0.03 and r = 0.21, p = 0.03, respectively). CCT did not correlate with axial length and refractive error (p ≥ 0.73).

**Table 1:** Characteristics and ORA measurements of the study population.

	Mean ± SD	Range
Age, y	23.7 ± 4.4	18 to 40
Spherical equivalent, D	−4.91 ± 2.03	−1.00 to −9.63
Axial length, mm	25.60 ± 1.04	22.62 to 28.77
Visual field mean deviation, dB	−2.10 ± 0.94	−4.58 to 1.47
CCT, μm	540.8 ± 29.0	444 to 612
CH, mmHg	9.77 ± 1.35	7.03 to 12.73
CRF, mmHg	9.57 ± 1.56	6.00 to 13.67
IOPcc, mmHg	15.80 ± 2.33	9.93 to 21.90
IOPg, mmHg	14.50 ± 2.64	7.63 to 21.30

CCT: central corneal thickness; CH: corneal hysteresis; CRF: corneal resistance factor; IOPcc: cornea-compensated intraocular pressure; IOPg: Goldmann-correlated intraocular pressure.

Table 2 demonstrates the mean measurements of optic nerve parameters and their relationship with CH and CCT. No significant correlation was detected between CH and RNFL measurements. For RNFL parameters obtained with OCT, there was a negative but not statistically significant correlation between CH and average RNFL thickness (r = −0.15, p = 0.13). With respect to SLP parameters, no significant relationship between CH and the RNFL parameters was detected. None of the tested disc parameters showed statistical significance with CH. For optic disc parameters measured with OCT, there was a negative but not statistically significant correlation between CH and rim area (r = −0.10, p = 0.29). CCT did not correlate with any of the RNFL measurements in OCT and SLP (Table 2). Positive but not statistically significant correlations between CCT and several disc measurements were found by using HRT (with r ranging from 0.11 to 0.17, all p ≥ 0.08, Table 2).

**Table 2:** Optic nerve parameters measured with 3 different imaging devices and their relationship with CH and CCT (Spearman’s correlation analysis, n = 108).

	Mean ± SD	CH		CCT	
		r (CI95%)	p	r (CI95%)	p
**SD-OCT**					
Average RNFL thickness, um	103.0 ± 8.5	−0.15 (−0.33 to 0.04)	0.13	−0.09 (−0.27 to 0.1)	0.33
Disc area, mm^2^	1.98 ± 0.50	−0.09 (−0.27 to 0.10)	0.37	0.11 (−0.08 to 0.29)	0.25
Rim area, mm^2^	1.49 ± 0.30	−0.10 (−0.28 to 0.09)	0.29	−0.06 (−0.25 to 0.13)	0.53
Cup-disc area ratio	0.43 ± 0.19	−0.02 (−0.21 to 0.17)	0.82	0.15 (−0.04 to 0.33)	0.11
Vertical cup-to-disc ratio	0.40 ± 0.19	−0.03 (−0.22 to 0.16)	0.75	0.17 (−0.02 to 0.35)	0.08
Cup volume, mm^3^	0.14 ± 0.15	−0.03 (−0.22 to 0.16)	0.77	0.14 (−0.05 to 0.32)	0.14
**GDx ECC**					
TSNIT average	57.93 ± 5.26	0.07 (−0.26 to 0.12)	0.47	0.02 (−0.17 to 0.21)	0.81
NFI	13.58 ± 7.80	−0.08 (−0.26 to 0.11)	0.40	−0.09 (−0.27 to 0.10)	0.37
TSNIT sd	29.33 ± 4.00	0.13 (−0.06 to 0.31)	0.17	0.02 (−0.17 to 0.21)	0.86
**HRT2 parameters**					
Disc area, mm^2^	1.92 ± 0.57	−0.01 (−0.18 to 0.20)	0.91	0.15 (−0.04 to 0.33)	0.13
Rim area, mm^2^	1.50 ± 0.30	−0.01 (−0.18 to 0.20)	0.93	0.14 (−0.05 to 0.32)	0.14
Cup-disc area ratio	0.19 ± 0.14	0.05 (−0.14 to 0.24)	0.59	0.15 (−0.04 to 0.33)	0.13
Vertical cup-to-disc ratio	0.28 ± 0.21	0.08 (−0.11 to 0.26)	0.39	0.13 (−0.06 to 0.31)	0.17
Cup volume, mm^3^	0.08 ± 0.12	0.03 (−0.16 to 0.22)	0.79	0.12 (−0.07 to 0.30)	0.22
Rim volume, mm^3^	0.51 ± 0.14	−0.11 (−0.29 to 0.08)	0.24	−0.04 (−0.23 to 0.15)	0.67
Mean cup depth, mm	0.20 ± 0.08	−0.02 (−0.21 to 0.17)	0.85	0.03 (−0.16 to 0.22)	0.74
Cup area, mm^2^	0.42 ± 0.42	0.03 (−0.16 to 0.22)	0.79	0.14 (−0.05 to 0.32)	0.14

CI: confidence interval; CH: corneal hysteresis; CCT: central corneal thickness.

Table 3 presents the subgroup analysis regarding the associations between CH and various optic nerve parameters in low to moderate myopia and high myopia. A similar pattern of correlations was observed. No significant relationship between CH and optic nerve parameters was detected (all p ≥ 0.10, Table 3) in both groups.

**Table 3:** Relationship between CH and optic nerve parameters measured with 3 different imaging devices in low to moderate myopia and high myopia (Spearman’s correlation analysis).

	Low to moderate myopia (n = 64)		High myopia (n = 44)	
	r (CI95%)	p	r (CI95%)	p
**SD-OCT**				
Average RNFL thickness, um	−0.12 (−0.35 to 0.13)	0.33	−0.21 (−0.47 to 0.09)	0.16
Disc area, mm^2^	−0.13 (−0.36 to 0.12)	0.33	0.07 (−0.23 to 0.36)	0.64
Rim area, mm^2^	−0.15 (−0.38 to 0.09)	0.25	0.08 (−0.22 to 0.37)	0.61
Cup-disc area ratio	−0.11 (−0.35 to 0.14)	0.41	0.10 (−0.20 to 0.38)	0.54
Vertical cup-to-disc ratio	−0.11 (−0.35 to 0.14)	0.38	0.07 (−0.23 to 0.36)	0.64
Cup volume, mm^3^	−0.12 (−0.35 to 0.13)	0.34	0.12 (−0.18 to 0.40)	0.45
**GDx ECC**				
TSNIT average	0.18 (−0.07 to 0.40)	0.16	0.18 (−0.12 to 0.45)	0.25
NFI	−0.20 (−0.42 to 0.05)	0.12	0.10 (−0.20 to 0.38)	0.53
TSNIT sd	0.21 (−0.43 to 0.04)	0.10	−0.12 (−0.40 to 0.18)	0.43
**HRT2 parameters**				
Disc area, mm^2^	−0.12 (−0.35 to 0.13)	0.35	0.22 (−0.08 to 0.48)	0.15
Rim area, mm^2^	−0.03 (−0.27 to 0.22)	0.83	0.13 (−0.17 to 0.41)	0.39
Cup-disc area ratio	−0.08 (−0.32 to 0.17)	0.51	0.21 (−0.09 to 0.48)	0.14
Vertical cup-to-disc ratio	−0.02 (−0.26 to 0.23)	0.89	0.23 (−0.07 to 0.49)	0.13
Cup volume, mm^3^	−0.11 (−0.35 to 0.14)	0.37	0.20 (−0.10 to 0.47)	0.18
Rim volume, mm^3^	−0.05 (−0.29 to 0.20)	0.70	−0.12 (−0.40 to 0.18)	0.45
Mean cup depth, mm	−0.11 (−0.35 to 0.14)	0.40	0.15 (−0.15 to 0.42)	0.33
Cup area, mm^2^	−0.11 (−0.35 to 0.14)	0.39	0.25 (−0.05 to 0.50)	0.10

CI: confidence interval.

## Discussion

Corneal biomechanics has been an area of much recent interest as a risk factor for glaucoma development and progression^1–3,5–7^. In the present study, we aimed to evaluate the relationship between CH and quantitative measurements of the RNFL and optic disc. By using all three devices, we did not detect any statistically significant relationship between CH and various optic nerve parameters in healthy myopic eyes.

Limited data regarding the association between CH and RNFL thickness has been reported in previous studies^9,18,19^. Bueno-Gimeno *et al*. found a positive correlation between CH and peripapillary RNFL thickness in 199 children^18^. Recently, in the EPIC-Norfolk Eye Study, CH was reported to positively correlate with RNFL measurement by using SLP technology in 5134 British subjects^9^. In contrast, Chang *et al*. reported that CH was not associated with peripapillary RNFL or macular inner retinal layer thickness in 100 myopic eyes of 50 myopic subjects by using spectral-domain OCT^19^. In the present study, we use both the SD-OCT and SLP to measure the RNFL thickness in healthy myopic eyes. Consistent with Chang’s study^19^, we did not find a significant relationship between CH and RNFL measurements in both devices. The discrepancy in the finding between these studies might partially be explained by differences in the study population, methodology and sample size. According to the methodology of the EPIC-Norfolk Eye Study, both healthy eyes and eyes with glaucomatous damage were included in the analysis^9^. Previous studies have shown that CH is significantly lower in glaucomatous eyes^3,5^. A positive correlation found between CH and RNFL measurement in their study might at least partially be explained by the effect of glaucoma damage on CH and RNFL. Moreover, it has to be noted that the correlation of CH to RNFL measurement shown in the EPIC-Norfolk Eye Study was rather weak (partial correlation coefficient = 0.106, p = 0.006)^9^, so a chance finding cannot be excluded, and the clinical implication of such a weak correlation remain to be confirmed.

Previous studies have evaluated the association between CH and optic disc parameters^8,19–21^. Lim *et al*. show that both CH and CRF were not associated with various optic disc parameters by using HRT 2 in 102 Singaporean children^20^. In a large cohort of 1645 healthy British twins, Carbonaro *et al*. reported that CH was not significantly correlated with measurements of optic disc cupping^8^. However, Chang *et al*. reported that both CH and CCT correlated negatively with several optic disc parameters by using SD-OCT in myopic subjects^19^. Of note, measurement error in OCT due to ocular magnification was not considered in Chang’s study. Previous studies have shown that ocular magnification in myopic eyes has important effect on the optic disc measurements^22^. In this study, the Littmann’s formula was used to adjust the ocular magnification for measurements obtained with OCT. In agree with most previous studies^8,20,21^, we did not find a significant association between CH and any of the optic disc parameters by using OCT and HRT technology.

The association between CCT and optic nerve parameters in healthy subjects has been reported in previous studies^23–25^. Although a rather weak correlation was detected between CCT and optic disc measurements in the Tajimi study^23^, most of the studies reported that CCT was not associated with the structural measurements of the optic nerve^24,25^. In concordance with the previous reports^24,25^, we found that CCT was not associated with any of optic nerve parameters measured with 3 imaging devices.

There are strengths in the present study. The comprehensive ocular examination in each subject permitted the exclusion of ocular pathologies (myopic chorioretinopathy, vitreomacular traction, myelinated retinal nerve fibres, peripapillary choroidal neovascularization and glaucoma) that are likely to influence the structural measurements of the optic nerve. All three imaging devices were performed on the same visit by the experienced technicians following a standardized protocol. Finally, various parameters were evaluated in correlation with corneal hysteresis. Our findings of no significant relationship between CH and optic nerve parameters were consistent across all three modalities, which reinforce the validity of these findings in the present study population.

However, there are also limitations in the present study. One limitation is that only young myopic subjects of the same ethnicity (all were Chinese) were included in the analysis. Thus, the current results may not apply to other populations. Another limitation is the cross-sectional nature of the present study. Thus, future longitudinal studies with large sample size are warranted to confirm our findings.

In conclusion, no statistically significant relationship was observed between CH and optic nerve parameters as measured by all three imaging modalities in healthy myopic eyes. Therefore, the relationship observed previously in glaucoma subjects is likely coming to fruition as optic nerve damage is caused by the disease.

## Methods

### Subjects

In this prospective, cross-sectional observational study, 116 Chinese healthy myopic subjects were consecutively recruited from the refractive surgery clinic of Joint Shantou International Eye Center. Each included subjects underwent a detailed ophthalmic examination including the measurement of refraction, visual acuity, intraocular pressure (IOP), central corneal thickness (CCT) measurement by an A-ultrasound pachymeter (Reichert Ophthalmic Instruments, Depew, NY, USA), axial length (IOL master; Carl-Zeiss Meditec, Dublin, CA), and a dilated stereoscopic fundus examination. All the included eyes had a spherical equivalent (SE) less than −0.50 diopters (D) and had no other concurrent ocular disease. One eye from each subject was chosen for analysis; if both eyes were eligible, a random eye was selected by using a computer programme^26^. Based on the refractive status, subjects were subdivided into two groups: high myopia group (SE ≤ −6.00 D) and low to moderate group (−6.00 D < SE ≤ −0.50). Subjects with best corrected visual acuity less than 20/40, contact lens use, IOP over 21 mmHg, family history of glaucoma, intraocular surgery, myopic macular degeneration, glaucoma, refractive surgery, neurological diseases or diabetes were excluded. The present study followed the tenets of the declaration of Helsinki and was approved by the local ethical committee. Written informed consent was obtained from each subject before enrolment.

### Visual field testing

Visual field testing was performed with the static automated white-on-white threshold 24-2 Swedish interactive threshold algorithm standard strategy (Humphrey Field Analyzer II; Carl Zeiss Meditec, Inc.). Only reliable visual field tests (with fixation loss, false positive and false negative were all less than 20%) were used in the study. All the included visual field tests were those with pattern standard deviation (PSD) with P > 5% and within normal limits in glaucoma hemifield test (GHT).

### Ocular Response Analyzer Measurement

Corneal hysteresis in each subject was performed with ORA (Reichert Ophthalmic Instruments, Depew, NY, USA), prior to CCT measurement. The details of the principles of the device have been described previously^4^. The device reports four parameters including corneal hysteresis (CH), corneal resistance factor (CRF), cornea-compensated intraocular pressure (IOPcc) and Goldmann-correlated intraocular pressure (IOPg). For each subject, the ORA examination was performed at least 3 times. The ORA software (Software Version: 2.02) reports a waveform score, ranging from 0 to 10, to ensure accurate measurement. All included ORA measurements had a waveform score no less than 5 in this study. Disqualified measurements (the waveform score less than 5) and irreproducible values were discarded and repeated measurements were performed. The average values of three measurements with desirable curves were recorded for subsequent analysis.

### Confocal Scanning Laser Ophthalmoscopy Imaging

Confocal scanning laser ophthalmoscopy (HRT 2; Heidelberg Engineering, GmbH, Dossenheim, Germany) was performed in all included eyes to obtain various optic disc parameters. After image acquisition, a single mean topography for analysis is generated by averaging three aligned consecutive scans. Image quality was checked carefully for all the optic disc images. All the contour lines were manually drawn by a trained ophthalmologist (KQ) and the disc margin was defined as the inner edge of the Elschnig’s ring. Only good quality images with an average pixel height standard deviation no more than 30 µm were included in the analysis. Global optic nerve head parameters including disc area, rim area, cup-to-disc ratio, cup volume and mean cup depth were used for analysis.

### Scanning Laser Polarimetry Imaging

Scanning laser polarimetry (SLP) imaging was performed by using GDx ECC algorithm (Carl Zeiss Meditec, Dublin, CA, USA). The eye-specific corneal birefringence consisting of the corneal polarization axis and magnitude was first determined for each subject. The RNFL was then quantified with GDx ECC algorithm (software version 5.5.0.14). Only images with an image quality check score no less than 8 were included. The raw data were exported from the instrument for subsequent analysis. The typical scan score (TSS) of each eye was collected to evaluate the atypical birefringence patterns. Only scans with a TSS no less than 80 were included in the present study^27^. The parameters of nerve fiber index (NFI), the temporal superior nasal inferior temporal (TSNIT) average RNFL measurement and TSNIT standard deviation (TSNIT-SD) were collected for subsequent analysis.

### Optical Coherence Tomography

Each of the included eye received optic disc imaging with the Cirrus High Definition OCT (Cirrus HD OCT, software version 5.0.0.326; Carl Zeiss Meditec, Dublin, CA) by using the Optic Disc Cube 200 × 200 protocol. The axial resolution for this spectral-domain OCT is 5 μm and the scan speed is 27000 A-scans per second. Scans with eye movements (with misaligned vessels within the scanning area) were excluded by reviewing the real-time SLO fundus images. All the included optic disc images had minimum signal strength of 7. The overall average RNFL thickness and optic disc parameters including disc area, rim area, average cup disc area ratio (ACDR) and vertical cup disc ratio (VCDR) were recorded from the analysis printout generated by Cirrus HD OCT.

### Adjustment for Ocular Magnification of Cirrus HD-OCT

According to previous studies, the relationship between the OCT measurement and its actual size can be expressed as *t* = *p* × *q* × *s*, where *t* is the actual fundus dimension, *p* is the magnification factor of the camera of the HD-OCT system, *s* the measurement obtained from the OCT system, and *q* the magnification factor of the eye^22^. For the HD-OCT system, *p* is known to be 3.382. The ocular magnification factor *q* of the eye can be calculated with the formula *q* = 0.01306 × (axial length − 1.82). Because *t* = *p* × *q* × *s* refers to linear magnification, the equation would be modified to *t* ^2^ = *p* ^2^ × *q* ^2^ × *s* ^2^ for both area and volume measurements (ocular magnification has no influence on the *z*-axis).

- To adjust area parameters (disc area and rim area), the following formula was used:
  Corrected area = 3.382^2^ × 0.01306^2^ × (axial length −1.82)^2^ × measured area
- As ocular magnification has no influence on the *z*-axis, the following formula was used to adjust volume parameters:
  Corrected volume = 3.382^2^ × 0.01306^2^ × (axial length−1.82)^2^ × measured volume
- To adjust average RNFL thickness measurement, the following formula was used:

Corrected average RNFL thickness = 3.382 × 0.01306 × (axial lengh −1.82) × measured average RNFL thickness

The corrected average RNFL thickness and optic disc measurements were used in the statistical analysis.

### Statistical Analysis

The statistical analyses were performed by using the SPSS software (ver. 17.0; SPSS Inc, Chicago, IL) and MedCalc software (ver. 12.1.4.0; Belgium). Spearman’s correlation analysis was performed to determine the effects of axial length/refractive error, CCT, and corneal biomechanics on various optic nerve parameters. Sample size calculation revealed that at least 85 eyes would be required to determine whether a correlation coefficient +/− 0.30 (a weak correlation) differs from zero with a statistical power of 80% at an alpha of 0.05. A *p* value less than 0.05 was considered statistically significant.
